# Psychosocial Reactions of Pakistani Students Towards COVID-19: A Prevalence Study

**DOI:** 10.12669/pjms.37.2.3063

**Published:** 2021

**Authors:** Zahid Mahmood, Sadia Saleem, Sara Subhan, Ayesha Jabeen

**Affiliations:** 1 Zahid Mahmood, PhD. Department of Clinical Psychology, under School of Professional Psychology, University of Management and Technology, Lahore, Pakistan; 2 Sadia Saleem, PhD. Department of Clinical Psychology, under School of Professional Psychology, University of Management and Technology, Lahore, Pakistan; 3 Sara Subhan, PhD. Department of Clinical Psychology, under School of Professional Psychology, University of Management and Technology, Lahore, Pakistan; 4 Ayesha Jabeen, Department of Clinical Psychology, under School of Professional Psychology, University of Management and Technology, Lahore, Pakistan

**Keywords:** Depression, Anxiety, COVID-19, Students

## Abstract

**Background:**

The current study aimed at investigating the manifestation and prevalence of the commonly reported psychosocial reactions in the university students following the onset of COVID-19 pandemic.

**Methods:**

This cross-sectional survey was carried out during April to May, 2020 in the city of Lahore. Based on the interviews and presenting problems of thirty-nine self-referred students to student counselling service center, a list of twenty-seven self-report measure was presented through an online cross-sectional survey of 510 students enrolled in a private institute. The age range of the participants was between 17-26 years (*M*, 21.86; *SD*, 2.94).

**Results:**

The results showed that the most frequently reported reactions by university students during COVID-19 is *restricted daily routine* (92%), *preoccupation with cleanliness* (86%), *feeling uncertain about future* (85%), *feeling bored* (84%) and *low mood* (84%). The least frequently reported reaction was *financial crisis* (48%). The findings further showed that 18% of the participants had *mild*, 34% had *moderate*, 29% *severe* and 19% *very severe* level of problems.

**Conclusions:**

The finding revealed that university students have significantly affected by this pandemic which need attention from mental health professionals.

## INTRODUCTION

The recent upsurge of fear, panic, and uncertainty caused by the rapid and dramatic spread of Coronavirus across all continents, countries, and communities has shaken our sense of safety and security.^1^ Corona virus reached in Pakistan by the end of February 2020 and complete lockdown was observed on 23^rd^ March, 2020 resulting in the closure of most of the industries and educational institutes.^2^ The magnitude of the problems caused by Corona-19 and its rates of spreading has caught us unprepared. We are all going through a difficult time emotionally, socially and educationally.^3^ University years are considered as one of the most crucial time periods in intellectual and psychological sense. University life demands extensive cognitive input, opportunity to make intimate relationships, explore career opportunities and meet expectations of parents.^4^ All these pressures requires a continuous phase of adjustment which may make them vulnerable to experience mental health issues. As performance of the students largely depends on their mental health therefore any deterioration may bring negative impacts to the individual and society.^5^ In 2011, a survey of mental health problems in a sample of 1850 university students of Lahore, Pakistan revealed that 31% of the participants fall in ‘’sever’’ category, and 16% fall in “very severe” category of psychological issues.^6^

In current circumstances, students are reeling under the impact of pandemic which is a topic of attention worldwide. Particularly, in underdeveloped countries like Pakistan where lack of facilities and training of online education system has challenged the stakeholders academically and emotionally.^3^ Traditional education system with face to face teaching tied student-teacher interaction in social and emotion bond which seemed threatened in an online education system.^7^ The impact of this calamity has attained greater attention from the researchers and been associated with various mental health consequences.^8^ The fear of this pandemic which is causing many deaths has significantly changed our lives, inculcated a sense of fear and dramatically restricted our personal and social lives to curtail its spread.^9^ University students were at different phases of their education when sudden lockdown happened. Some were ready to appear in their final examination, some just started their semester, and other were ready to go to the field to get practical exposure.^10^ The uncertainty about the prevailing situation geared up stress in students. Apprehensions about online education system, financial crisis and concerns of parents about the future of their children was an add-on to stress.^11^

Researches have shown that lack of personal space at home, imposed social distancing with class mates, inability to interact with peers, friends and teacher’s in-person, limited access to educational resources, led the students to experience boredom and frustration.^7^,^8^ A paradigm shift in education system due to pandemic anticipated uncertainties about future in students. A plethora of research evidence revealed that chronic illness may aggravate mental health problems like depression, anxiety and posttraumatic stress.^12^ The available literature reveals that anxiety, depression, stress, poor sleep, and suicidal ideation are the most common psychological problems reported by general people and professionals.^13^,^14^

The psychological states caused by sudden, unexpected and frightening experiences can often lead to clouding of critical faculties by bias, prejudice, irrational attitudes that in turn is likely to worsen the problem.^15^ Therefore, it is very pertinent to explore the experience and expression of psychological reactions developed by students, who not only been feeling panicky and fearful but also had to undergo restricted social activities and social lockdown which eventually exacerbate their suffering and symptoms of distress.^16^ Such experiences limit their resources to cope with this pandemic. The early identification of people at risk of serious mental health problems can facilitate timely intervention.^17^

The current research, therefore, aimed to explore the commonly reported psychosocial reactions of university students in this widespread outbreak of COVID-19 so that tailor-made psychological intervention can be planned and provided timely to manage and cope with the adverse consequences of COVID-19.

## METHODS

A sample of 510 graduate students (men 39.60% and 60.40% women) participated through an online survey studying in a private university of city Lahore (Pakistan) during April to May, 2020. The age range was between 17-26 years with mean age 21.86+ 2.94 participated in the survey Table-I. Participants being unmarried, having access to internet and with ability to understand English was included in the study using purposive sampling technique.

**Table-I:** Frequency and Percentage of Demographic Variables of Participants (N=510)

*Variables*	*f*	*%*
***Age***		
18-21	273	53.50
22-27	237	46.50
***Gender***		
Men	202	39.60
Women	308	60.40
***Class***		
BS1	90	17.60
BSII	93	18.20
BSIII	58	11.40
BSIV	102	20.00
MS I & II	167	33.01
***Family System***		
Nuclear	347	68.00
Joint	163	32.00

***Note:***f:Frequency, %:Percentage

Total 344 participants (68%) belonged to nuclear family system and 161 (32%) belonged to joint family system. Most of the parents of the participants had high level of education and only about 30% had minimum education up to Middle level. Approval from Institutional Ethical Committee was taken (IRB No. 2019-05-010, dated 03-05-2020) Written informed consent was taken from participants through email, they were ensured about confidentiality, anonymity and were given right to withdraw during interview. The measure of the current study comprised a Demographic Form reporting age, gender, educational level and the living system. A self-report likert-type measure, the Psychosocial Reaction Scale (PRS) comprising 27 items based on the presenting problems of 39 self-referred students at the Counselling Service Center during COVID-19 lockdown period in Pakistan. The response options include 0 (*not at all*), 1(*a little*), 2(*to some extent*), and 3(*a lot*). Participants were asked to rate each statement to the extent to which it applies to them. The scoring range is between 0 to 81, higher score denotes to more psychosocial reactions experienced by university students. The Cronbach alpha for the current research is .92 showing a high internal consistency of PRS. Results were analyzed by using IBM SPSSV21.0, World cloud was used to highlight the themes from verbatim given in interviews of students.

## RESULTS

**Fig.1:**
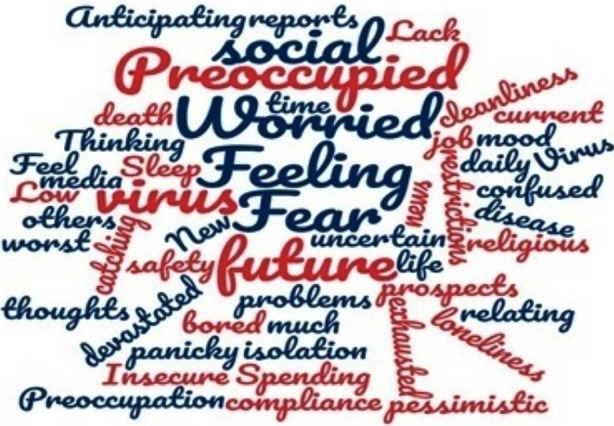
Pictorial Description of Psychosocial Reactions Experienced by Students during COVID-19.

A pictorial description of the frequently reported psychosocial reactions expressed by university students is presented in Fig.1.

The prevalence rate of psychosocial reactions was determined by deriving the participants’ total score on PSR. The sample was divided into four categories of severity of according to means and the standard deviations in the following manner. These four categories are mild (1 *SD* below the mean), moderate (mean), severe (1 *SD* above the mean) and very severe (2 *SD* above the mean).

Table-I shows that restricted daily routine, preoccupation with cleanliness, feeling uncertain about the future and low mood, boredom, anticipating the worst news were at the top of the list. Table-II

**Table-II:** Frequency of most Frequently Reported Psychosocial Reactions (N=510).

*Item No*	*Items*	*f*	*%*
23	Restricted daily routine	471	92
22	Preoccupied with cleanliness	439	86
5	Feeling uncertain about the future	436	85
3	Feeling bored	428	84
7	Low mood	428	84
18	Anticipating the worst news	427	84
24	Worried about job prospects	421	83
27	Spending more time on social media reports	421	83
4	Fear of catching the disease	414	81
1	Feeling confused	403	79
8	Feeling pessimistic about the future.	387	76
14	Lack of compliance by others	381	75
26	Feeling exhausted	384	75
15	Fear of loneliness	375	74
19	Feeling devastated	378	74
2	Insecure	362	71
10	Worried about own safety	364	71
11	Feeling aches and pains	364	71
17	Worried about the future	362	71
20	Fear of death	347	68
25	Preoccupied with "what if…"	348	68
6	Sleep problems	346	67
9	Feeling panicky	339	66
13	Feel social isolation	314	62
15	Preoccupation with thoughts relating to the current virus	280	55
21	Becoming more religious	250	49
12	Financial crisis	247	48

### Prevalence of Psychosocial Reactions

The prevalence of psychosocial reactions was determined by deriving the participants’ total score on PSR. The sample was divided into four categories of severity of according to means and the standard deviations in the following manner. These four categories are mild (1 *SD* below the mean), moderate (mean), severe (1 *SD* above the mean) and very severe (2 *SD* above the mean).

**Fig.2:**
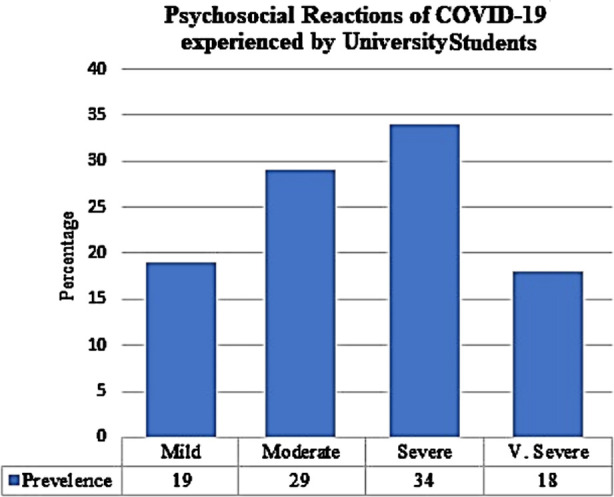
Distribution of the Participants (N = 510) on Severity of PSPS Score.

However, Independent sample *t*-test showed non-significant results with respect to gender and family system in their experience of psychosocial reactions.

## DISCUSSION

The pandemic of COVID-19 spread so quickly that world was not prepared and ready to understand it. Until very recently, very few mental health professionals had undertaken serious scientific studies to understand the psychological impact of COVID-19 and a limited research is available to understand the magnitude of the psychosocial reactions experienced and expressed by this unpredictable situation. Pakistan is an under developing country that has limited health care facilities, low education and lack of awareness have created more panic and fear of the situation and sudden lockdown and social distancing have created stress and anxiety.^8^

Contrary to other correlational studies, the current study is an attempt to identify the nature, pattern, and magnitude of the psychosocial problems experienced by university students as a result of COVID-19. The first consideration of the current research was to use tailor-made assessment procedures rather than frequently used anxiety and depression measures developed in other contexts and on another set of samples. We used an open-ended approach by collating a list of the most frequently reported reactions of the self-referred individual in the counselling service. This gives us a contextually and ecologically valid yardstick to measure the psychological impact of COVID-19. The overall picture of the presenting problem is in line with the existing literature.^1^,^9^ These problems give a picture of depressive features, a sense of apprehensions, and fear of unknown, panic and so on.

Results of the current study have revealed that the topmost problem reported by students is *restricted daily routine* which represents the major hurdle of university years.^3^ During normal circumstances, university students have a very active personal, academic and social life that also plays the role of a coping mechanism to handle daily life stressors and having to adjust with a new way of life.^14^ The second most frequently reported problem was the *preoccupation with cleanliness*; bearing in mind that cleanliness has a very special function in our culture in everyday life where they have to wash their hands before and after every meal and to perform ablution five times a day. This is also because of a constant campaign through social media to raise awareness about the precautionary measures to prevent oneself from COVID-19. The third most frequently reported problem is *feeling uncertain about the future* indicates a sense of apprehensions and anxiety .This may be because of the lack of knowledge about the disease and not having any tangible remedy specifically used for COVID-19. Contrary to western literature, financial issues doesn’t appear very prominently because in Pakistani context, usually parents are responsible for children’s education often at graduate and postgraduate level.^4^

The prevalence rate is alarming in nature and a large number of university students have been suffering from mental health concerns.^18^ Almost half the respondent need psychological first aid in one way or the other. It is important to note that in a short time our life has been turned upside down, changing our view of ourselves and the world. From a relatively safe and predictable living, we are now afraid to think of our future. Such anxieties are a natural reaction because of the fact of how events unfolded. At a time of sudden and severe stress, we observe two extreme reactions.^19^,^20^ Some overreact with panic measures and others deny the danger and perhaps pretend nothing is going to happen to them. Most of us move from one reaction to the other^21^. This situation of uncertainty arises especially when we do not have enough information to form a realistic view of the problem and our reaction. There is a great need for comprehensive and cohesive need-based services to address the arising state of confusion and uncertainty. Future research also focused on different segments of populations so the mental health services can be expanded.

### Limitations of the study

The study represented the experiences of students who had availability of internet, smart phones and email addresses. However, it may not be representative of the issues of students having lack of such facilities.

## CONCLUSIONS

The results of the study revealed that COVID-19 pandemic affected students cognitively, behaviorally and socially. The psychological symptoms measured by indigenous measure reflected high prevalence of these issues indicating the dire need of intervention.

### Authors Contribution:

**ZM:** Supervised the project and is responsible and accountable for the accuracy or integrity of the work.

**SS:** Prepared the manuscript and enhanced conceptual understanding

**SS:** Prepared the google doc, carried out initial statistical analysis, data collection

**AJ:** Provided statistical support and also reviewed the document.
